# Mutation update for the *ACTN2* gene

**DOI:** 10.1002/humu.24470

**Published:** 2022-09-27

**Authors:** Johanna Ranta‐aho, Montse Olive, Marie Vandroux, Giorgia Roticiani, Cristina Dominguez, Mridul Johari, Annalaura Torella, Johann Böhm, Janina Turon, Vincenzo Nigro, Peter Hackman, Jocelyn Laporte, Bjarne Udd, Marco Savarese

**Affiliations:** ^1^ Folkhälsan Research Center Helsinki Finland; ^2^ Department of Medical Genetics, Medicum University of Helsinki Helsinki Finland; ^3^ Department of Neurology, Neuromuscular Diseases Unit Hospital de la Santa Creu i Sant Pau Barcelona Spain; ^4^ Biomedical Research Institute Sant Pau (IIB Sant Pau) Barcelona Spain; ^5^ Centro de Investigación Biomédica en Red de Enfermedades Raras (CIBERER) Madrid Spain; ^6^ IGBMC (Institut de Génétique et de Biologie Moléculaire et Cellulaire) Université de Strasbourg Illkirch France; ^7^ Department of Neurology, Neuromuscular Unit, Hospital Universitario 12 de Octubre, Research Institute imas12, Biomedical Network Research Centre on Rare Diseases (CIBERER) Instituto de Salud Carlos III Madrid Spain; ^8^ Department of Precision Medicine University of Campania ‘Luigi Vanvitelli’ Naples Italy; ^9^ Department of Neurology Vaasa Central Hospital Vaasa Finland

**Keywords:** *ACTN2*, alpha‐actinin‐2, cardiomyopathy, congenital myopathy, distal myopathy

## Abstract

*ACTN2* encodes alpha‐actinin‐2, a protein expressed in human cardiac and skeletal muscle. The protein, located in the sarcomere Z‐disk, functions as a link between the anti‐parallel actin filaments. This important structural protein also binds N‐terminal titins, and thus contributes to sarcomere stability. Previously, *ACTN2* mutations have been solely associated with cardiomyopathy, without skeletal muscle disease. Recently, however, *ACTN2* mutations have been associated with novel congenital and distal myopathy. Previously reported variants are in varying locations across the gene, but the potential clustering effect of pathogenic locations is not clearly understood. Further, the genotype‐phenotype correlations of these variants remain unclear. Here we review the previously reported *ACTN2*‐related molecular and clinical findings and present an additional variant, c.1840‐2A>T, that further expands the mutation and phenotypic spectrum. Our results show a growing body of clinical, genetic, and functional evidence, which underlines the central role of *ACTN2* in the muscle tissue and myopathy. However, limited segregation and functional data are available to support the pathogenicity of most previously reported missense variants and clear‐cut genotype‐phenotype correlations are currently only demonstrated for some *ACTN2*‐related myopathies.

## 1 INTRODUCTION

**Figure 1:**
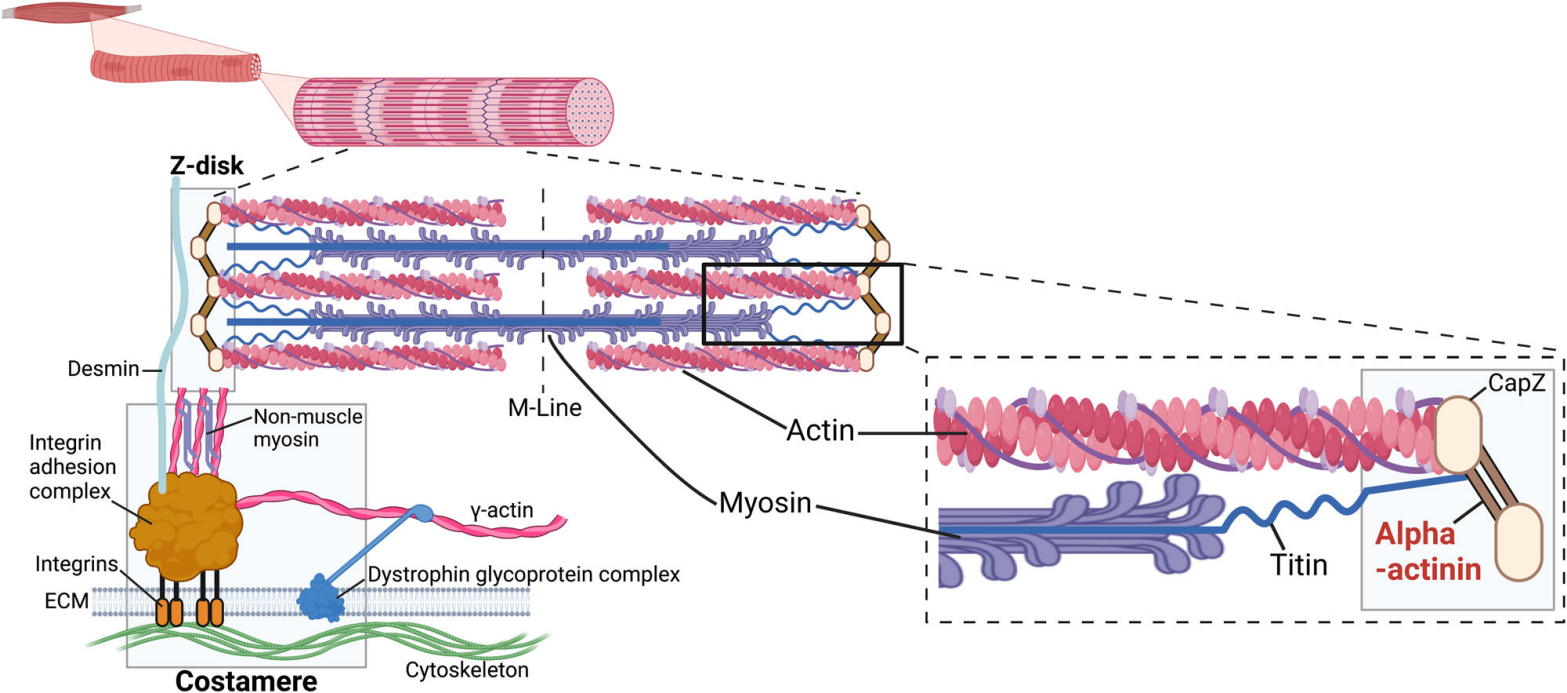
Representation alpha‐actinin location in the sarcomere. Created with BioRender.

Human alpha‐actinins are highly conserved proteins, which are classified into four different main isoforms, coded by four distinct genes (Murphy & Young, 2015). Nonsarcomeric isoforms, alpha‐actinin‐1 and 4, link actin filaments in the cytoskeleton, whereas alpha‐actinin 2 and alpha‐actinin 3 are major components of the sarcomeric Z‐disk (Figure 1) (Beggs et al., 1992; Murphy & Young, 2015; Ribeiro Ede et al., 2014). Alpha‐actinin‐2 is expressed in both skeletal and cardiac muscle, while alpha‐actinin‐3 is only expressed in skeletal muscle. A single‐nucleotide polymorphism, that causes alpha‐actinin‐3 deficiency, is common in humans. Thus, the deficit of alpha‐actinin‐3 isoform is not pathogenic, likely due to high compensatory expression of alpha‐actinin‐2 in individuals with this alpha‐actinin‐3 deficiency (Berman & North, 2010; Gupta et al., 2012). Contrarily, several studies suggest that defects in alpha‐actinin‐2, coded by *ACTN2* located on chromosome 1q43, may result in skeletal and cardiac muscle diseases. More specifically, variants in *ACTN2* and subsequent alterations in alpha‐actinin‐2 structure or expression have been previously associated with several types of cardiomyopathies (hypertrophic, dilated, and arrhythmogenic), with or without left ventricular compaction (Bagnall et al., 2014; Chiu et al., 2010). Recently, *ACTN2* variants have also been associated with congenital and adult‐onset distal myopathies (Lornage et al., 2019; Savarese et al., 2019, 2021).

**Figure 2:**
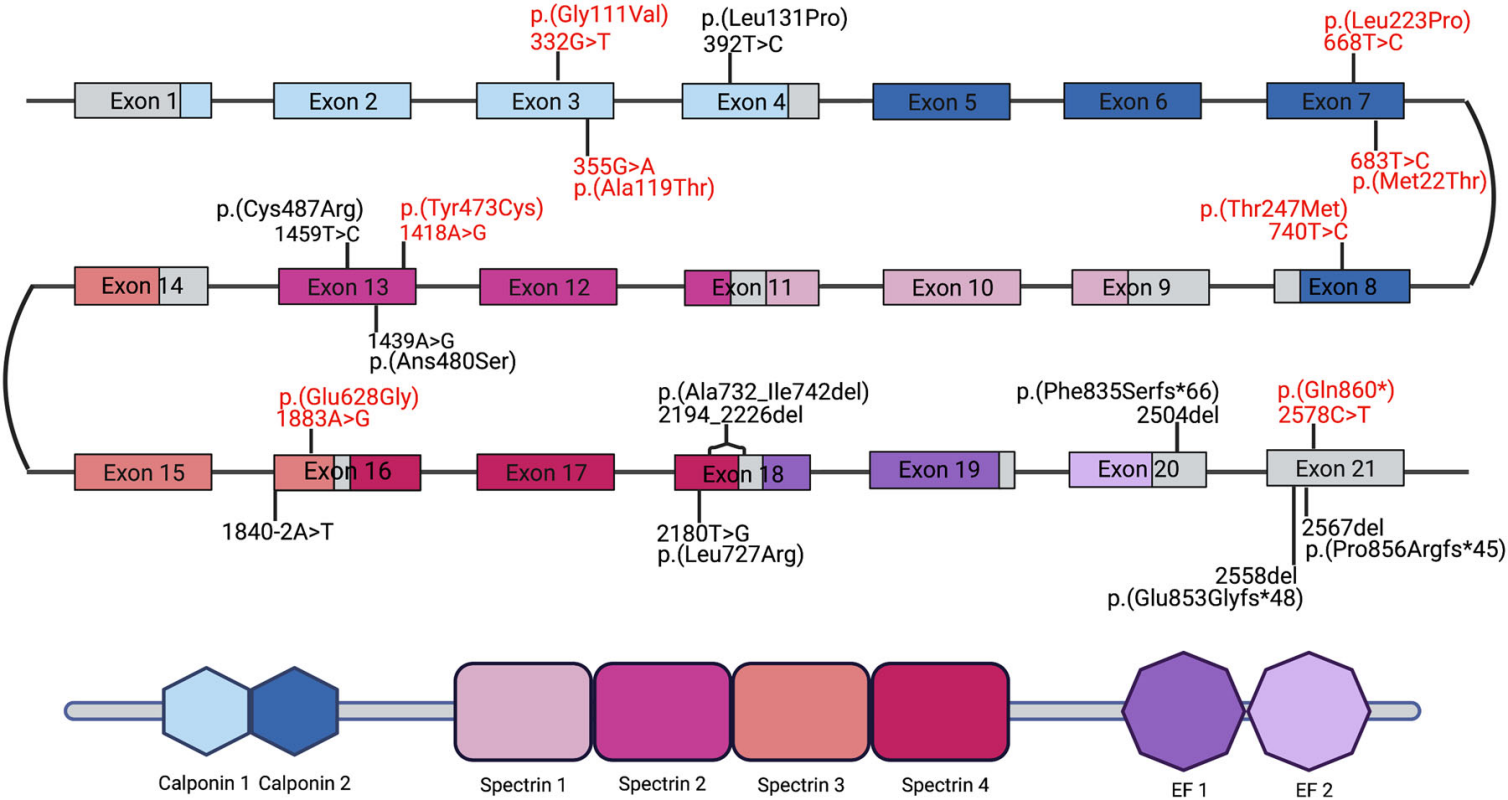
Schematic representation of *ACTN2* gene structure with corresponding protein domains, and the locations of pathogenic point mutations along the gene. Gene regions and their respective protein domains are indicated by matching colors. Variants associated with cardiomyopathy are shown in red, while mutations associated with skeletal muscle disease are shown in black. Large deletions and insertions are not shown. Representation is not in scale. Created with BioRender.

Structurally, alpha‐actinin‐2 includes an N‐terminal actin‐binding domain (ABD) containing two calponin homology domains (CH1 and CH2), a central rod domain with four spectrin‐like repeats, and a C‐terminal calmodulin‐like domain (CAMD) with two EF‐hand motifs (Beggs et al., 1992; Ribeiro Ede et al., 2014) (Figure 2). Alpha‐actinin‐2 binds the actin filaments via its N‐terminal ABD. In contrast, C‐terminal CAMD fixates titin to the Z‐disk through binding with titin's N‐terminal Z‐repeats (Grison et al., 2017; Young & Gautel, 2000). This interaction is highly dynamic and probably regulated by phospholipids and other intramolecular mechanisms (Grison et al., 2017; Ribeiro Ede et al., 2014; Young & Gautel, 2000). Alpha‐actinin‐2 functions as a mechanosensor and a signaling hub for other Z‐disk proteins, many of which are known to cause distal myopathies when mutated (Murphy & Young, 2015; Ribeiro Ede et al., 2014; Savarese et al., 2020; Young & Gautel, 2000).

Here, we provide an overview of the reported *ACTN2* cases, and review the current knowledge of the mutation impact and molecular pathomechanisms. We also describe novel *ACTN2* variants and thereby expand the clinical and mutational spectrum of *ACTN2*‐related disorders (actininopathies).

## 2 MATERIALS AND METHODS

### 2.1 Database query and literature search

We reviewed and updated the *ACTN2* page on the Leiden Open Variation Database (LOVD V.3.0) to include, at the best of our knowledge, all the *ACTN2* variants previously reported in literature.

We also reviewed the *ACTN2* variants identified in previously unpublished, large resequencing projects that were conducted at the Telethon Institute of Genetics and Medicine (TIGEM, Italy), at the Institut de Génétique et de Biologie Moléculaire et Cellulaire (IGBMC, France), and at the Folkhälsan Research Center (FRC, Finland). The TIGEM cohort included 844 index patients, mainly with abnormality of the central nervous system, skeletal muscle diseases, or eye diseases. The patients were exome sequenced within a national program for undiagnosed patients, the Telethon Undiagnosed Program. The IGBMC Myocapture resequencing project included 1170 exomes from patients with congenital myopathies (96%), or other muscular dystrophies or neuropathies (4%), and their healthy relatives. Finally, the FRC cohort comprised of over 2000 patients with skeletal muscle diseases, mostly with an adult‐onset distal myopathy, who were analyzed using a targeted gene panel (Evila et al., 2016). All patients and their relatives provided written informed consent.

For each variant, we collected available clinical and familial segregation data. The pathogenicity of the variants was evaluated based on the guidelines provided by American College of Medical Genetics and Genomics and the Association for Molecular Pathology (ACMG/AMP) (Richards et al., 2015) using Varsome clinical version 11.3.5 (Kopanos et al., 2019). The variants were classified in the five suggested categories according to the available evidence.

We carefully evaluated the strength of the PVS1 criteria to be applied to loss of function variants, using AutoPVS1, an automatic classification tools for PVS1 use (Xiang et al., 2020). Similarly, we carefully evaluated the application of PP5 (reputable sources reporting the variant as pathogenic) and PM5 (novel missense change at an amino acid residue where a different missense change determined to be pathogenic has been seen before). All the variants were reported using the reference transcript NM_001103.3.

### 2.2 Characterization of the *ACTN2* c.1840‐2A>T variant

We performed a segregation analysis on the available family members (primers available on request). For splicing characterization, we extracted RNA from the muscle biopsy (tibialis anterior) using the Qiagen RNeasy Plus Universal Mini Kit (Qiagen) according to the manufacturer's instructions. Strand‐specific RNAseq library was prepared using the NEBNext Ultra II Directional RNA Library Prep Kit for Illumina (New England Biolabs), and sequenced on HiSeq. 4000 (Illumina), generating over of ~40 million reads (75 bp paired‐end sequencing). Trimmed sequences were mapped with STAR 2.7.0d (Dobin et al., 2013) using the two‐pass method. Gene level read summarization and quantification was done by featureCounts (Liao et al., 2014). To characterize the splicing defect due to the previously detected nucleotide change, we compared *ACTN2* spliced reads (“junctions”) in the patient with an internal control group (RNAseq data from over 200 myopathy patients and over 30 non myopathy controls). In addition, sashimi plots from the index patient were manually inspected using the Integrative Genomics Viewer (IGV) for the *ACTN2* gene.

## 3 GENOTYPES AND PHENOTYPES

### 3.1 Variants identified in patients with a skeletal muscle disorder

Five studies reported eight causative *ACTN2* variants in patients with a skeletal muscle disorder, including seven heterozygous mutations with de novo occurrence or dominant disease inheritance, and one homozygous mutation with recessive disease transmission (Table 1).

**Table 1:** Previously described variants in patients with skeletal muscle actininopathy

DNA change (NM_001103.3) and zygosity	GnomAD v2.1.1 ALL frequency	GnomAD POPmax frequency	Varsome prediction and criteria	Supporting data	Phenotype	Reference
c.392T>C (p.Leu131Pro) het	NA	NA	VUS+ (PM2 strong, PP3 supp)	Segregation in a single family	Distal myopathy (mainly lower legs)	Savarese et al. (2019)
c.1439A>G (p.Asn480Ser) hom	0.000003977	NFE:0.000003977	VUS+ (PM2 strong)	Segregation in multiple families	Distal myopathy (mainly lower legs) leading to a generalized weakness	Inoue et al. (2021)
c.1459T>C (p.Cys487Arg) het	NA	NA	VUS+ (PM2 strong, PP3 supp)	Segregation in multiple families	Distal myopathy (mainly lower legs)	Savarese et al. (2019)
c.2180T>G (p.Leu727Arg) het	NA	NA	LP (PM2 strong, PP3 supp, PM6 moderate)	Functional data	Congenital myopathy	Lornage et al. (2019)
c.2194_2226del p.(Ala732_Ile742del) het	NA	NA	LP (PM2 strong, PP3 supp, PM6 moderate)	NA	Congenital myopathy	Lornage et al. (2019)
c.2504del p.(Phe835Serfs*66) het	NA	NA	LP (PVS1 moderate, PM2 strong)	Segregation in a single family and functional studies	Distal myopathy (mainly lower legs) with a later involvement of proximal muscles	Chen et al. (2021)
c.2558del p.(Glu853Glyfs*48) het	NA	NA	LP (PVS1 moderate, PM2 strong)	Segregation in a single family	Distal myopathy (mainly lower legs) with facial muscle weakness	Savarese et al. (2021)
c.2567del p.(Pro856Argfs*45) het	NA	NA	LP (PVS1 moderate, PM2 strong)	Segregation in a single family	Distal myopathy (mainly lower legs) with facial muscle weakness	Savarese et al. (2021)

Abbreviations: het, heterozygous; hom, homozygous; LP, likely pathogenic; NA, not available; NFE, European (non‐Finnish); supp, supporting evidence; VUS, variant of uncertain significance; VUS+, VUS with minor pathogenic evidence.

Two heterozygous de novo variants, p.Leu727Arg and p.Ala732_Ile742del, absent in the genome aggregation database (gnomAD), were identified in patients with congenital myopathy (Lornage et al., 2019). The phenotype included facial muscle weakness and ophthalmoplegia, ptosis, and high palate. Creatinine Kinase (CK) levels were normal. The histological analysis and electron microscopy imaging of muscle biopsies showed muscle fibers containing small structured cores and jagged Z‐lines. A single patient showed cardiac insufficiency in the neonatal period that disappeared following treatment with corticosteroids and digitalins.

A heterozygous variant, p.Cys487Arg, absent in gnomAD, was described in 15 patients from northern Spain with a late‐onset distal myopathy (from 40 to 60 years old). The muscle weakness began in the anterior compartment of the lower limbs with a subsequent involvement of posterior lower leg and proximal lower limb muscles (Savarese et al., 2019). Another heterozygous missense variant, p.Leu131Pro, reported in the same study, was present in two patients with a similar phenotype. All patients showed walking difficulties. In all patients, CK levels were elevated, particularly in the early stages of the disease. Muscle biopsies showed rimmed vacuolar pathology and sarcomeric abnormalities. Magnetic resonance imaging (MRI) findings reflected the disease stage with early involvement of tibialis anterior muscles and extensor digitorum longus, soleus, and gastrocnemius medialis. In all these patients, cardiac function was normal.

In three patients from two families, recently, two different heterozygous single nucleotide deletions (c.2558del and c.2567del) were identified (Savarese et al., 2021). These deletions, seen in the last exon of the ACTN2, were predicted to result in an elongated protein (p.Glu853Glyfs*48 and p.Pro856Argfs*45, respectively). Slowly progressive weakness of the distal lower limb and facial muscles was observed in all patients. Muscle pathology included internalized nuclei, myofibrillar disorganization, and rimmed vacuoles. Muscle MRI showed complete fatty replacement of anterolateral compartment muscles of the lower legs without involving thigh muscles. A single patient showed dilated cardiomyopathy.

Three patients showed a heterozygous frameshift variant causing a protein extension (p.Phe835Serfs*66) (Chen et al., 2021). These patients presented with somewhat varying symptoms, resembling those in the patients with a similar protein extension described by Savarese and colleagues. All patients experienced distal lower limb weakness but in varying severity. Two patients presented with bilateral foot drop with steppage gait, while the third patient experienced more severe weakness in the proximal upper limbs.

In addition to the heterozygous variants, a single homozygous variant, p.Asn480Ser, was identified in four Japanese patients from three unrelated families (Inoue et al., 2021). The patients presented with late‐onset (ranging from 30 to 60 years old) muscle weakness beginning in distal lower legs, which evolved towards generalized muscle weakness. The muscle pathology report indicated increased fibers with internal nuclei in all patients, minicore‐like structures, and rimmed vacuoles. CK was normal or slightly increased. Muscle MRI showed complete fatty replacement of the posterior compartment muscles of the thighs, tibialis anterior, and medial head of the gastrocnemius, while the anterior compartment of the thigh was spared. Myocardial diastolic dysfunction and atrial fibrillation were reported in a single patient at 78 years.

### 3.2 Variants identified in patients with an isolated cardiomyopathy

Many unique, rare *ACTN2* variants have been identified in patients with cardiac diseases. These cardiac conditions included hypertrophic cardiomyopathies, dilated cardiomyopathies, and arrhythmias, without any reported skeletal muscle involvement. The variants associated with these conditions have been described in various clinical reports and/or identified in many large resequencing studies. Following the ACMG/AMP guidelines, we reclassified 10 of these variants as pathogenic or likely pathogenic (Table 2), as sufficient evidence was demonstrated since segregation and/or functional data are available.

**Table 2:** Previously described pathogenic or likely pathogenic variants in patients with cardiomyopathy

DNA change (NM_001103.3) and zygosity	GnomAD v2.1.1 ALL frequency	GnomAD POPmax frequency	Varsome prediction and criteria	Supporting data	Phenotype	Reference
c.332G>T (p.Gly111Val) Het	0.00005657	NFE:0.0001084	LP (PM2 strong, PP3 supp, PP5 supp, PS3 supp)	Functional data	HCM	Theis et al. (2006); Haywood et al. (2016)
c.355G>A (p.Ala119Thr) Het	NA	NA	P (PP5 very strong, PM2 strong, PP3 supp)	Segregates in multiple families	Variable symptoms including LVNC, DCM, and ventricular fibrillation often resulting in cardiac arrest	Bagnall et al. (2014); Chiu et al. (2010); Isbister et al. (2021); Kraoua et al. (2022)
c.668T>C (p.Leu223Pro) Het	NA	NA	VUS+ (PM2 strong, PP3 supp)	Segregates in a single family	LVNC	Park et al. (2021)
c.683T>C (p.Met228Thr) Het	NA	NA	VUS+ (PM2 strong, PP3 supp)	Segregates in a single family	Arrhythmia (paroxysmal supraventricular tachycardia/atrioventricular block)	Girolami et al. (2014)
c.740C>T (p.Thr247Met) Het	NA	NA	LP (PM2 strong, PP3 supp, PP3 moderate)	Segregates in a single family	Varying degrees and types of arrhythmias (supraventricular and ventricular); HCM observed in three patients	Prondzynski et al. (2019)
c.1418A>G (p.Tyr473Cys) Het	NA	NA	VUS+ (PM2 strong, PP3 supp)	Segregates in a single family member	Left dominant ACM observed in three patients	Good et al. (2020)
c.1883A>G (p.Glu628Gly) Het	NA	NA	VUS+ (PM2 strong, PP3 supp)	Segregates in a single family	Mild to moderate left ventricular hypertrophy	Burns et al. (2017); Chiu et al. (2010)
c.2578C>T (p.Gln860*) Hom	NA	NA	VUS+ (PM2 moderate, PVS1 moderate)	Functional data	Progressive heart failure symptoms, episodes of atrial fibrillation, and restrictive cardiomyopathy	Lindholm et al. (2021)
g.236898807_236903093del insTTCTTCAGCCAGTCCCATTGGCTCTTCTTCCAGAATACATC; c.698_1107delinsTTCTTCAGCCAGTCCCATTGGCTCTTCTTCCAGAATACATC (p.Asp233_Ser369delinsValLeuGlnProValProLeuAlaLeuLeuProGluTyrIle) Het	NA	NA	NA	Segregates in a single family + functional data	Varying degrees and types of arrhythmias (supraventricular and ventricular); LVNC in four patients; cardiac arrest in two cases	Lindholm et al. (2021)
chr1:236881685_236891006del; c.241+413_565del p.? Het	NA	NA	NA	Segregates in a single family	HCM observed in two patients, LVNC in a single patient	Singer et al. (2021)

Abbreviations: ACM, arrhythmogenic cardiomyopathy; DCM, dilated cardiomyopathy; func, functional studies supporting pathogenicity; HCM, hypertrophic cardiomyopathy; het, heterozygous; hom, homozygous; LP, likely pathogenic; LVNC, left ventricular noncompaction cardiomyopathy; NA, not available; NFE, European (non‐Finnish); P, pathogenic; VUS, variant of uncertain significance; VUS+, VUS with minor pathogenic evidence; (a relative with DCM also carries the pathogenic variant OR a relative without phenotype does not carry the pathogenic variant).

A heterozygous *ACTN2* variant, p.Ala119Thr (not in gnomAD), was found in a total of 17 patients (Bagnall et al., 2014; Chiu et al., 2010; Isbister et al., 2021; Kraoua et al., 2022). The conditions observed varied from mild cardiac symptoms to cardiac arrest.

Another heterozygous variant, p.Met228Thr (not listed in gnomAD), was described in 11 patients. However, clinical data were available for only nine patients (Girolami et al., 2014). All patients presented with arrhythmia (paroxysmal supraventricular or atrial tachycardia, atrioventricular block, or premature atrial contraction). Comparable phenotypes were observed in the four patients with the p.Thr247Met variant (Prondzynski et al., 2019). All patients presented with varying degrees and types of arrhythmias, including left bundle branch block, atypical flutter, paroxysmal atrial fibrillation or supraventricular tachycardia, as well as atrial, ventricular, or supraventricular premature beats. In addition, three out of the four patients suffered from HCM. Similarly, a heterozygous variant p.Glu628Gly was reported in three patients presenting with HCM or mild hypertrophy (Burns et al., 2017; Chiu et al., 2010).

The heterozygous p.Tyr473Cys variant was reported in four patients (Good et al., 2020). Left dominant arrhythmogenic cardiomyopathy (ACM) was diagnosed in three out of the four patients. These patients also showed right bundle branch block morphology, ventricular premature beats, dyspnea, and palpitations. A left ventricular noncompaction cardiomyopathy with decreased ejection fraction was observed in three patients carrying a heterozygous p.Leu223Pro variant (Park et al., 2021).

Further, three unique *ACTN2* variants (p.Gly111Val, p.Thr495Met, and p.Arg759Thr) were found in three different patients (Theis et al., 2006). The patients with the p.Thr495Met and p.Gly111Val variants both presented with HCM. The pathology report showed endocardial fibrosis in both cases and myocyte disarray in the patient with the latter variant. Functional evidence supports the pathogenicity of p.Gly111Val (Haywood et al., 2016), whereas multiple studies suggest that p.Thr495 is a benign variant (Akinrinade et al., 2015; Andreasen et al., 2013; Chiu et al., 2010; Gonzalez‐Garay et al., 2013; Verdonschot et al., 2020). No pathology report or further evidence of pathogenicity is available for the patient with the p.Arg759Thr variant that is, thereby, classified as VUS.

A heterozygous, large deletion at the position chr1:g.236881685_236891006del of *ACTN2* was found in three individuals (Singer et al., 2021). The variant, a 9.3 kb intragenic deletion, spans from intron 2 to exon 6 of *ACTN2*. Two patients from the same family presented with HCM, whereas the third patient was diagnosed with left ventricular noncompaction. A heterozygous inframe indel (a deletion at the position g.236898807_236903093—spanning exons 8 through 10—and a 41 bp long intronic insertion) was reported in 11 patients (Lindholm et al., 2021). The inframe indel caused a truncated protein of 771 amino acids, compared to the wild‐type *ACTN2* (894 amino acids long). Out of the 11 patients, 9 patients had clinical data available. These patients presented with various cardiac symptoms; however, eight out of the nine patients experienced some form of arrhythmia, including atrial fibrillation and flutter, supraventricular tachycardia, right bundle branch block, and premature ventricular contractions. Another common finding was left ventricular noncompaction, which was observed in four patients. The cardiac phenotype of many of these patients was quite severe, as four patients experienced heart failure symptoms, including cardiac arrest in two cases.

Multiple heterozygous variants identified in patients with cardiac diseases were described in clinical reports suggesting their possible clinical impact, although later studies did not confirm the pathogenicity. A heterozygous p.Gln9Arg variant, for example, was documented in a patient diagnosed with dilated cardiomyopathy (Mohapatra et al., 2003). However, later studies suggest that this variant is benign (Andreasen et al., 2013; Cuenca et al., 2016; Nouhravesh et al., 2016; Pugh et al., 2014).

In addition to the heterozygous variants, two unique homozygous variants were recently described. A single patient was reported to carry the nonsense variant p.Gln860* (Lindholm et al., 2021). This patient developed progressive heart failure symptoms, episodes of atrial fibrillation, and restrictive cardiomyopathy before undergoing heart transplantation surgery at the age of 23. A homozygous variant, p.Thr412Met, was also found in a single case with sonographically identified fetal pleural effusions. However, the clinical significance of this variant is uncertain (Jelin et al., 2020).

Finally, many additional *ACTN2* variants have been identified in large sequencing projects focused on cardiac diseases (Supporting Information: Table S1). Some of these variants may have clinical relevance, however, further clinical, genetic (segregation) and functional data are needed to properly assess their clinical significance. On the other hand, some other variants can be classified as benign or likely benign, based on, for example, high frequency of the variant in the population.

Interestingly, several truncating variants (nonsense or indels causing a frameshift and a premature stop codon and splice variants) have been reported in heterozygosity in patients with a cardiac phenotype. Although these variants are of interest (e.g., some of them are classified as likely pathogenic variants), we still lack sufficient evidence to classify them as pathogenic since haploinsufficiency has not been proved as a disease mechanism.

### 3.3 Animal models and functional validation of causative variants

Alpha‐actinin‐2 function has been previously studied in zebrafish (Gupta et al., 2012). Knockdown experiments using splice‐site‐blocking and translational morpholinos showed reduced movement and behavior anomalies of the morphant fish compared to the wild type, suggesting an overall muscle weakness in the morphants. A closer examination of the phenotype revealed that the morphants had smaller eyes, enlarged hearts with a reduced heartbeat, and abnormal organization of skeletal muscles.

Further, histological analysis and electron microscopy imaging of the morphant skeletal and cardiac muscle were conducted. They showed small, disorganized fibers, often lacking typical striations, and containing many centrally localized, round nuclei. The histological analysis showed no evidence of dystrophic processes in the affected muscle fibers, suggesting that alpha‐actinin‐2 deficiency would lead to defective sarcomerogenesis at the early stages of development.

The histological analysis and electron microscopy imaging of the cardiac muscle of the *ACTN2* morphants showed atrial and ventricular dilation, with markedly thin walls. In addition, the size and number of sarcomeric assemblies were significantly reduced in the cardiomyocytes of the morphant fish.

A thorough functional characterization of the p.Leu727Arg variant was performed by Lornage and colleagues (Lornage et al., 2019). The results from modeling the disease in zebrafish and mice by exogenous expression of p.Leu727Arg‐mutated *ACTN2* were consistent with the abnormal muscle function and structure seen in the patients. In addition, motor defects were observed in zebrafish, and the reduction of muscle force was noted in isolated muscles from AAV‐transduced mice. In both models, sarcomeric disorganization was apparent, whereas the exogenous expression of wild‐type alpha‐actinin‐2 had no pathogenic effect on the skeletal muscle structure.

Lindholm and colleagues extensively characterized a nonsense variant p.Gln860* and the aforementioned indel variant resulting in a truncated protein of 771 amino acids. Using patient pluripotent stem cell‐derived cardiomyocytes (hiPSC‐CMs), they showed a decreased contractile velocity of single hiPSC‐CMs, but normal Ca^2+^ signaling and sarcomere structure. The study also found that the nonsense variant p.Gln860* produces a truncated protein lacking its C‐terminus, which causes the loss of interaction with sarcolemma‐associated proteins, such as alpha‐actinin‐1 and gap junction protein alpha 1. The authors concluded that this variant represents a hypomorphic variant, which causes a recessive disease with healthy carriers. The inframe indel was found to produce a shortened protein that is expressed and incorporated in the Z‐disk with a possible dominant‐negative effect.

Chen and colleagues characterized their reported variants in C2C12 myocytes. It was demonstrated that, in transiently transfected C2C12 cells, the p.Leu131Pro and p.Phe835Serfs*66 variants resulted in aggregates of alpha‐actinin‐2 (Chen et al., 2021). Similarly, the p.Gln9Arg mutation was reported to affect actinin nuclear localization in cultured cells (C2C12), simultaneously inhibiting the initiation of cellular differentiation (Mohapatra et al., 2003). However, the high frequency of the mutations in the population suggests that this variant is benign (Supporting Information: Table S1).

Finally, circular dichroism and X‐ray crystallography studies demonstrated the p.Gly111Val and p.Ala119Thr variants, identified in HCM patients, cause small but relevant changes in the secondary and tertiary structure of alpha‐actinin‐2. Both variants also impaired the Z‐disk localization of actinin and its binding to F‐actin (Haywood et al., 2016).

#### 3.3.1 Variants in TIGEM, IGBMC, and FRC resequencing projects

Fourty‐nine rare *ACTN2* variants identified in three different resequencing projects have been re‐evaluated to identify possible novel variants of interest (Supporting Information: Table S2).

One variant, p.Gly111Arg, associated with hypertrophic cardiomyopathy has been identified in a patient with a skeletal muscle disease and some heterozygous variants may have a possible clinical impact (e.g., p.Ser369Trp or p.Arg492*). Their clinical assessment is still pending.

**Figure 3:**
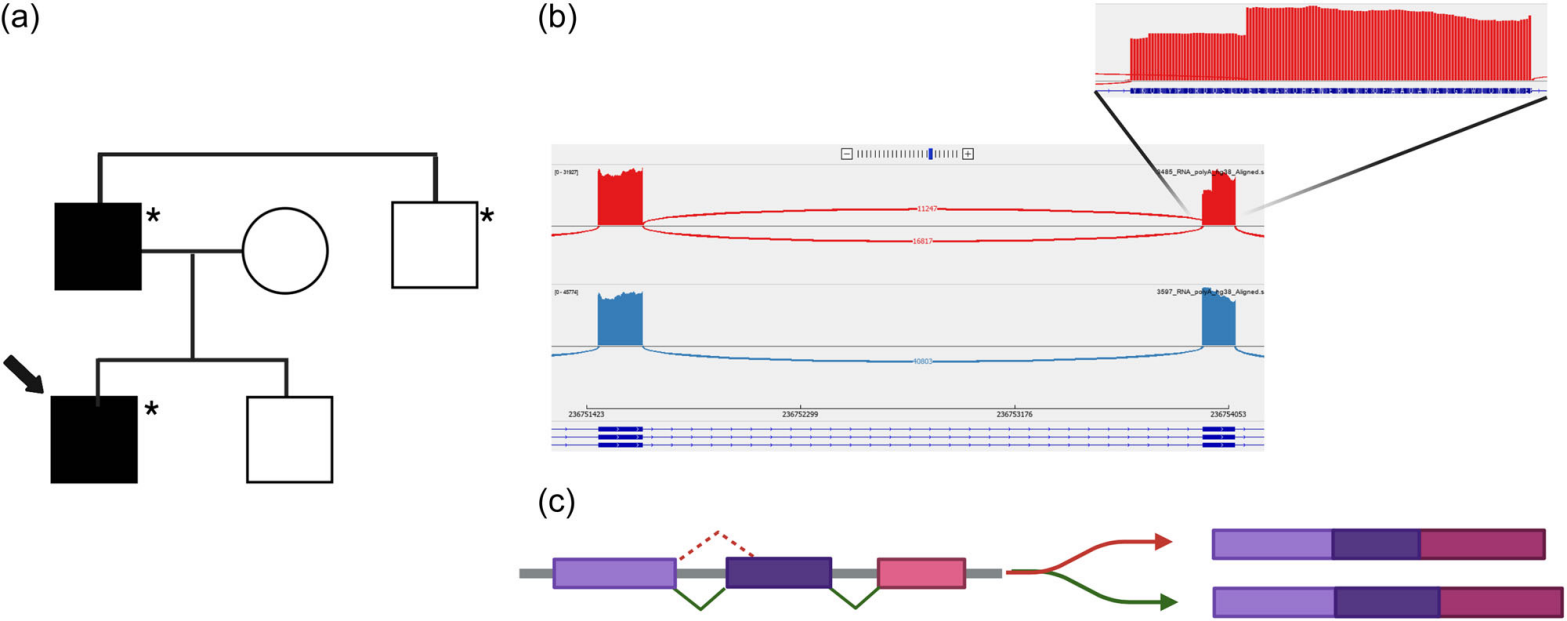
(a) Pedigree of the family characterized by the variant c.1840‐2A>T. An asterisk (*) denotes an individual genetically tested for the presence of the variant. (b) Sashimi plot showing the variant splice junctions from aligned RNA‐seq data with a magnification showing the loss of the first 39 nucleotides of exon 16. (c) Schematic representation of the alternative splicing pattern of the variant c.1840‐2A>T. Created with BioRender.

Interestingly, a Spanish patient with clinical presentation, an adult‐onset distal myopathy, and muscle pathology resembling the previously described adult‐onset actininopathy patients, harbored a variant affecting a canonical splice site (c.1840‐2A>T). The variant segregated with the disease in the family (Figure 3a). Analysis of cDNA derived from the proband's muscle biopsy demonstrated the activation of an alternative acceptor site and the in‐frame deletion of 39 nucleotides with the consequent skipping of 13 amino acids (c.1840_1878del p.(Val614_Gln626del)) (Figure 3b,c).

Finally, a homozygous missense variant (p.Asn101Ser) was identified in a French patient with congenital myopathy. Segregation and functional studies of this variant are ongoing.

## 4 CONCLUSIONS

In this study, to the best of our knowledge, we have listed all the *ACTN2* variants previously reported in literature. Of these variants, eight variants (four missense, an inframe deletion and three deletions causing a protein extension) have been demonstrated to cause a skeletal muscle disease (Figure 2). Similarly, ten variants (two large deletions, seven missense changes in heterozygosity and one nonsense in homozigosity) reach enough evidence to be classified as causative of a cardiac disease (Table 2).

Limited segregation and functional data are currently available to support the pathogenicity of most previously reported missense variants in patients with a cardiac disease. Similarly, the clinical impact of heterozygous loss of function variants is still far from being exhaustively characterized. According to the the ClinGen expert panel for HCM, only moderate evidence supports the gene‐disease association between *ACTN2* and cardiomyopathies (Clinical Genome Resource, 2018). Moreover, the variable cardiac presentations, including cardiomyopathies and arrhythmias, could all be included under the umbrella‐term intrinsic cardiomyopathy. The possible role of modifier genetic and nongenetic factors adds a further level of complexity in the interpretation of the pathogenicity of *ACTN2* variants associated with cardiomyopathy. On the other hand, the association between *ACTN2* mutations and myopathy is supported by a mounting body of clinical, genetic, and functional evidence.

### 4.1 Genotype‐phenotype correlations

We do not have a clear understanding of why some variants result in isolated cardiomyopathy and others in skeletal muscle disease with or without heart involvement. Out of all the patients with skeletal muscle actininopathy, only a few patients show overt cardiac involvement. The later development of cardiac symptoms in patients affected explicitly by skeletal muscle disease should not be excluded and a regular cardiac follow up would be needed. It is also possible that some milder muscle weakness could be overlooked in patients with cardiac disease.

Moreover, there is limited correlation between the location of the variants to specific domains or structures of the protein and the cardiac or skeletal phenotype. The three indels found in the last two exons, which are predicted to produce an extended protein, cause a very similar phenotype with a combined weakness of the distal lower leg and facial muscles in all identified patients (Chen et al., 2021; Savarese et al., 2021). Interestingly, recessive forms of actininopathies have been recently described (Inoue et al., 2021; Lindholm et al., 2021). Biallelic actininopathies do not seem to cause more severe disease than the dominantly inherited forms.

#### 4.1.1 Diagnostic and clinical implications

The interpretation of rare *ACTN2* variants is challenging, and thus it is essential to carefully apply the ACMG/AMP guidelines (Richards et al., 2015) to avoid a misclassification. In particular, we suggest that a single heterozygous missense change should not be reported as causative if the classification is not supported by large segregation and functional data. Similarly, further data is needed to demonstrate that haploinsufficiency is a disease mechanism and leverage the classification of heterozygous loss‐of‐function variants.

Moreover, based on our findings, it is currently not possible to identify clear hallmarks of *ACTN2*‐related cardiac and skeletal myopathy. Thus, we suggest that variant interpretation should be performed cautiously. Finally, we recommend that a regular cardiac follow up should be administrated for patients with a genetically confirmed dominant or recessive skeletal muscle actininopathy, since the later development of cardiac symptoms cannot be excluded.

#### 4.1.2 Future prospects

Further functional and mechanistic data would be needed to provide definitive proof of pathogenicity for some of the previously reported variants and to address the still‐unsolved questions regarding the pathomechanisms underlying the different forms of actininopathies.

## CONFLICT OF INTEREST

The authors declare no conflict of interest.

## Supporting information

Supporting information.

Supporting information.

## Data Availability

The data that support the findings of this study are available in Leiden Open Variation Database at https://databases.lovd.nl/shared/genes/ACTN2
